# Machine Learning Model for Predicting Coronary Heart Disease Risk: Development and Validation Using Insights From a Japanese Population–Based Study

**DOI:** 10.2196/68066

**Published:** 2025-05-12

**Authors:** Thien Vu, Yoshihiro Kokubo, Mai Inoue, Masaki Yamamoto, Attayeb Mohsen, Agustin Martin-Morales, Research Dawadi, Takao Inoue, Jie Ting Tay, Mari Yoshizaki, Naoki Watanabe, Yuki Kuriya, Chisa Matsumoto, Ahmed Arafa, Yoko M Nakao, Yuka Kato, Masayuki Teramoto, Michihiro Araki

**Affiliations:** 1 Artificial Intelligence Center for Health and Biomedical Research, National Institutes of Biomedical Innovation, Health and Nutrition, 3-17 Senrioka-shinmachi, Osaka, 566-0002, Japan, 81 8093069457; 2 NCD Epidemiology Research Center, Shiga University of Medical Science, Shiga, Otsu, Japan; 3 Department of Cardiac Surgery, Cardiovascular Center, Cho Ray Hospital, Ho Chi Minh City, Vietnam; 4 Department of Preventive Cardiology, National Cerebral and Cardiovascular Center, Suita, Osaka, Japan; 5 Libyan Centre for Dental Research, Zliten, Libya; 6 Faculty of Informatics, Yamato University, Osaka, Japan; 7 Department of Cardiology, Center for Health Surveillance and Preventive Medicine, Tokyo Medical University Hospital, Tokyo, Japan; 8 Department of Public Health, Faculty of Medicine, Beni-Suef University, Beni-Suef, Egypt; 9 Division of Health Sciences, Osaka University Graduate School of Medicine, Suita, Osaka, Japan; 10 Graduate School of Medicine, Kyoto University, Kyoto, Japan; 11 Graduate School of Science, Technology and Innovation, Kobe University, Kobe, Japan

**Keywords:** coronary heart disease, machine learning, logistic regression, random forest, support vector machine, Extreme Gradient Boosting, Light Gradient-Boosting Machine, Shapley Additive Explanations, CHD, SVM, XGBoost, LightGBM, SHAP

## Abstract

**Background:**

Coronary heart disease (CHD) is a major cause of morbidity and mortality worldwide. Identifying key risk factors is essential for effective risk assessment and prevention. A data-driven approach using machine learning (ML) offers advanced techniques to analyze complex, nonlinear, and high-dimensional datasets, uncovering novel predictors of CHD that go beyond the limitations of traditional models, which rely on predefined variables.

**Objective:**

This study aims to evaluate the contribution of various risk factors to CHD, focusing on both established and novel markers using ML techniques.

**Methods:**

The study recruited 7672 participants aged 30-84 years from Suita City, Japan, between 1989 and 1999. Over an average of 15 years, participants were monitored for cardiovascular events. A total of 7260 participants and 28 variables were included in the analysis after excluding individuals with missing outcome data and eliminating unnecessary variables. Five ML models—logistic regression, random forest (RF), support vector machine, Extreme Gradient Boosting, and Light Gradient-Boosting Machine—were applied for predicting CHD incidence. Model performance was evaluated using accuracy, sensitivity, specificity, precision, area under the curve, *F*_1_-score, calibration curves, observed-to-expected ratios, and decision curve analysis. Additionally, Shapley Additive Explanations (SHAPs) were used to interpret the prediction models and understand the contribution of various risk factors to CHD.

**Results:**

Among 7260 participants, 305 (4.2%) were diagnosed with CHD. The RF model demonstrated the highest performance, with an accuracy of 0.73 (95% CI 0.64‐0.80), sensitivity of 0.74 (95% CI 0.62‐0.84), specificity of 0.72 (95% CI 0.61‐0.83), and an area under the curve of 0.73 (95% CI 0.65‐0.80). RF also showed excellent calibration, with predicted probabilities closely aligning with observed outcomes, and provided substantial net benefit across a range of risk thresholds, as demonstrated by decision curve analysis. SHAP analysis elucidated key predictors of CHD, including the intima-media thickness (IMT_cMax) of the common carotid artery, blood pressure, lipid profiles (non–high-density lipoprotein cholesterol, high-density lipoprotein cholesterol, and triglycerides), and estimated glomerular filtration rate. Novel risk factors identified as significant contributors to CHD risk included lower calcium levels, elevated white blood cell counts, and body fat percentage. Furthermore, a protective effect was observed in women, suggesting the potential necessity for gender-specific risk assessment strategies in future cardiovascular health evaluations.

**Conclusions:**

We developed a model to predict CHD using ML and applied SHAP methods for interpretation. This approach highlights the multifactor nature of CHD risk evaluation, aiming to support health care professionals in identifying risk factors and formulating effective prevention strategies.

## Introduction

Coronary heart disease (CHD) remains a leading cause of morbidity and mortality worldwide, responsible for approximately 9.14 million deaths in 2019 [1,2]. Early identification of individuals at high risk is crucial, as timely interventions can significantly reduce the likelihood of severe outcomes like heart attacks and strokes. Studies have shown that early prediction and intervention can lead to a notable reduction in CHD-related mortality through preventive treatments such as statins and lifestyle changes [1-3]. While conventional risk assessment models have been used, there is growing recognition of the potential of machine learning (ML) in enhancing CHD prediction [4,5].

ML algorithms have proven their ability to analyze complex data and identify intricate patterns and relationships that are not easily detected by traditional statistical methods [6-10]. By integrating diverse data sources, such as demographics, medical history, lifestyle habits, and diagnostic findings, these algorithms can predict the likelihood of developing CHD. This approach offers comprehensive risk evaluation, adaptability to new data, and the potential to uncover novel risk factors and disease mechanisms [11].

Several studies have demonstrated the effectiveness of ML models in deriving quantitative markers for coronary artery disease and predicting the presence of heart disease. For example, a study developed and validated a coronary artery disease–predictive ML model using electronic health records and assessed its probabilities as in silico scores for coronary artery disease in participants in 2 longitudinal biobank cohorts [12]. Another study applied an ensemble ML model for coronary disease prediction, using ML classifiers to predict heart disease [13]. These findings highlight the potential of ML in driving innovation and improving the accuracy of CHD diagnosis and prediction [14].

However, challenges exist in utilizing ML for CHD prediction, including data quality, feature selection, model interpretability, and generalizability. These issues must be carefully addressed to ensure the reliability and robustness of the predictive models. Rigorous validation, regulatory compliance, and effective communication strategies are essential for its successful integration into clinical practice.

While several established CHD prediction models rely on traditional statistical techniques with predefined risk factors, they are limited by linear assumptions and struggle with complex, high-dimensional datasets. This restricts their ability to uncover novel or subtle risk factors. In contrast, ML models can handle these complexities, offering more nuanced and accurate predictions by identifying nonlinear interactions and discovering previously overlooked factors. Therefore, ML may enhance the overall understanding of CHD and improve both risk assessment and prevention strategies.

This study aimed to address the role of ML techniques in predicting incident CHD and identifying novel risk factors. This study sought to deepen our understanding of the factors contributing to CHD development by analyzing a comprehensive dataset. These findings will enhance risk assessment, enabling the development of personalized interventions and preventive strategies.

## Methods

### Study Design and Participants

The Suita Study, a prospective population-based cohort study, was conducted in Suita City, Osaka, Japan. From 1989 to 1999, a total of 7672 men and women aged 30-84 years who did not have a previous history of cardiovascular disease were recruited for the study. Participants were selected from the population registry of the municipality and were followed up every 2 years for an average of 15 years until their first occurrence of stroke, myocardial infarction (MI), death, or relocation.

After excluding participants with missing outcome data and removing unnecessary variables, the analysis included 7260 participants and 28 variables. Opt-out procedures were implemented for those who preferred not to participate in this study. Informed consent was obtained from all participants at the time of enrollment. The study followed the Transparent Reporting of a Multivariable Prediction Model for Individual Prognosis or Diagnosis and Artificial Intelligence (TRIPOD+AI) statement guidelines for reporting prediction models in medicine, and we have added the completed checklist in Checklist 1 [15].

### Ethical Considerations

The study was conducted in compliance with the ethical standards outlined in the Declaration of Helsinki, and approval was granted by the Institutional Review Board at the National Cerebral and Cardiovascular Center (approval R21024-2). As this study involves secondary data analysis, it is important to note that the original informed consent, obtained during the primary data collection, permits the use of the data for secondary analyses without requiring additional consent from participants. Participants’ privacy was protected by anonymizing or deidentifying the data to prevent identification.

### Outcome

The primary outcome was CHD, including MI, sudden death within 24 hours of acute illness onset, and coronary artery disease requiring bypass surgery or intervention. Medical records were carefully reviewed by hospital doctors or researchers who were blinded to the baseline data to provide an unbiased approach to the analysis. MIs were classified as definite or probable according to the criteria established by the MONICA Project [16].

Every 2 years, each participant’s health was evaluated at the National Cerebral and Cardiovascular Center in Osaka, Japan, to detect the occurrence of CHD. Yearly questionnaires were also completed by all participants by mail or telephone. CHD surveillance was completed by systematically searching for death certificates [17,18].

### Predictors

Predictors were measured at baseline and processed according to a standardized protocol. A comprehensive and prospective data collection process was implemented, encompassing various aspects such as demographics, medical history, medical imaging, laboratory data, lifestyle habits, and outcomes.

#### Blood Pressure and Physical Measurements

Blood pressure was measured in each participant using a mercury column sphygmomanometer, an appropriately sized cuff, and a standardized protocol to ensure accuracy and precision [17]. Before the initial blood pressure reading, the participants were instructed to rest for at least 5 minutes to establish a stable baseline. Blood pressure readings were obtained by averaging the second and third measurements, which were performed at intervals of more than 1 minute to allow for adequate observation and recording. Hypertension was defined as systolic blood pressure ≥140 mm Hg, diastolic blood pressure ≥90 mm Hg, or the use of antihypertensive medications.

BMI was calculated as weight (kg) divided by the square of height (m^2^).

#### Biochemical Measurements

At baseline, routine blood tests were conducted, including measurements of total cholesterol, high-density lipoprotein cholesterol (HDL-c), and fasting glucose levels. Non-HDL-c was calculated by subtracting HDL-c from total cholesterol. Diabetes mellitus was diagnosed if participants had fasting plasma glucose (FPG) ≥126 mg/dL, a non-FPG ≥200 mg/dL, or the use of diabetes mellitus medication.

The estimated glomerular filtration rate (eGFR; mL/min/1.73 m^2^) was calculated according to the original Modification of Diet in Renal Disease equation modified by the Japanese coefficient (0.881) as follows: 0.881×186×serum creatinine^−1.154^ × age^−0.203^ × (0.742 if female) [19].

#### Imaging Diagnostics

Carotid artery measurements were performed using a high-resolution ultrasound machine to assess atherosclerotic indices, specifically intima-media thickness (IMT), on both sides of the common carotid artery (CCA), carotid artery bulb, internal carotid artery, and external carotid artery. The maximum IMT in the CCA (IMT_cMax) was defined as the highest measurable IMT in the scanned CCA regions, while the maximum IMT (IMT_MAX) was the highest measurable IMT across the entire scanned area, including the CCA, bulb, internal carotid artery, and external carotid artery on both sides [20].

Atrial fibrillation was checked by standard 12-lead ECGs from all participants and was determined by well-trained physicians [18].

#### Lifestyle and Medical History

Smoking status and drinking statuses were categorized as current, quit, or never. A questionnaire was used to ask participants about their past and present history of CHD.

### Sample Size

All available data were used, and no formal sample size calculation was performed. The dataset included 7260 participants, among whom 305 had CHD, with 28 predictors selected after the feature selection process used in the model. Based on the events per predictor ratio, which is approximately 10.89 (305/28), the sample size is sufficient to ensure model stability and reliability [21,22]. Therefore, this dataset is adequate to answer the research questions.

### Missing Data

Missing data analysis was conducted, and variables with more than 30% missing values were excluded to enhance model robustness. Missing data were imputed using Multivariate Imputation by Chained Equations. See Multimedia Appendix 1 for details on the percentage of missing data for each variable before imputation.

### Statistical Analysis Methods

#### Descriptive Analysis

Continuous variables were summarized using means and SDs for normally distributed data, or medians and IQR for nonnormally distributed data. Categorical variables were reported as frequencies and percentages. To compare differences in patient characteristics based on CHD incidence (yes or no), we used various statistical tests including 2-tailed Student *t* tests, Mann‒Whitney *U* tests, or chi-square tests, as appropriate.

#### Feature Selection

Feature selection was conducted in a stepwise manner to ensure that only the most relevant variables were included in the predictive models. Initially, variables with more than 30% missing data were excluded to avoid potential bias from imputation. Following this, a correlation matrix was used to identify and remove variables with high multicollinearity, defined as having a correlation coefficient greater than 0.8. See correlation coefficients heat map in the Multimedia Appendix 2 for details. The next step involved applying the least absolute shrinkage and selection operator regression. This technique shrinks the coefficients of less significant predictors toward zero, effectively removing them from the model, and was performed using cross-validation to identify the most important features based on the data. Finally, after statistical feature selection, medical knowledge was applied to confirm the clinical relevance of the remaining variables. Important predictors such as age, glucose levels, HDL-c, and blood pressure were retained, given their established association with CHD. The list of variables (predictors) used for model development was described in Multimedia Appendix 3.

### Development of ML Models

#### Overview

The goal of this analysis was to predict the incidence of CHD using ML models and examine the contribution of each risk factor to the CHD incidence. A comprehensive process was followed, which included descriptive analysis, feature selection, model training, hyperparameter optimization, and interpretability through Shapley Additive Explanation (SHAP) values.

To manage the imbalance between CHD and non-CHD cases, we used down sampling on the majority class (non-CHD) to create a balanced dataset. This approach helps to ensure that the models do not disproportionately favor the majority class during training, improving prediction performance on the minority class.

The dataset was split into training (80%) and testing (20%) sets while maintaining balanced target variable distributions across both. Next, one-hot encoding was applied to convert categorical variables into a binary format, and normalization was performed to scale numerical features.

Several ML algorithms were implemented to compare their predictive power. Logistic regression (LR) was used as a baseline model, offering simplicity and interpretability [23]. Random forest (RF), an ensemble learning method, was used due to its strength in handling high-dimensional data and offering feature-importance insights [8,24]. Support vector machines (SVMs) with radial basis kernels were used for their effectiveness in nonlinear classification tasks [25,26]. Extreme Gradient Boosting (XGBoost) is an ML algorithm that improves model performance by using a series of decision trees, where each tree corrects the mistakes of the previous one. This sequential approach helps make predictions more accurate. Light Gradient-Boosting Machine (LightGBM) is another efficient algorithm that works similarly to XGBoost but is designed to be faster and more scalable, especially when working with large datasets and many features. Both algorithms are known for their high performance in handling complex data and large-scale problems [9,27].

#### Model Evaluation

We used 5-fold cross-validation during model training to ensure robustness and mitigate overfitting. Hyperparameter optimization was conducted using a grid search approach. The model’s performance on the testing set was evaluated using 5 metrics: accuracy, sensitivity, specificity, precision, area under the curve (AUC), and *F*_1_-score [15].

Calibration plots are used to evaluate the predictive accuracy of ML models in estimating CHD incidence. Calibration measures how closely the predicted absolute risk corresponds to the observed (true) risk across groups of patients categorized into different risk levels. The overall observed-to-expected (OE) ratio is calculated by dividing the total observed events by the total expected events for the entire population. For each decile, the OE ratio is determined by dividing the observed events within that decile by the expected events for the same decile. An ideal model is represented by a straight line bisecting the calibration plot, with an OE ratio of 1, indicating perfect calibration. An OE ratio <1 suggests overprediction, while a ratio >1 indicates underprediction [15].

Decision curve analysis (DCA) assesses the clinical use of ML models for predicting CHD incidence. DCA uses net benefit as a metric, reflecting the tradeoff between true-positive and false-positive predictions for a specific strategy [15,28,29].

#### Model Interpretation

SHAP is a method used in ML to make the predictions of a model more understandable. It helps explain how each input feature (such as age, cholesterol levels, or blood pressure) affects the model’s decision. Essentially, SHAP breaks down the prediction to show how much each feature contributes to the final result, allowing us to see which factors are most important for predicting a condition like CHD [8-10,30]. SHAP summary plots visualized the importance of key features, while SHAP dependence plots highlighted the non-linear relationships between features and CHD incidence.

## Results

### Study Participants’ Characteristics

In this study, 7260 participants were analyzed, of which 305 (4.2%) were diagnosed with CHD. The median age of participants with CHD was 63 (IQR 56-71) years , which was significantly older than that of those without CHD, whose median age was 55 (IQR 44-65) years. CHD was more prevalent in men (n=202, 66.2%) compared to women (n=103, 33.8%), and this gender difference was statistically significant.

Several cardiovascular risk factors were also associated with CHD. Participants with CHD had higher systolic and diastolic blood pressures. The eGFR was lower in participants with CHD compared to those without. The IMT of CCAs, IMT_cMax, was also significantly higher in patients with CHD (1.10 mm vs 1.00 mm; *P*<.001).

BMI and waist circumference were also higher in participants with CHD, indicating a greater degree of obesity. Additionally, lipid profiles showed significant differences, with lower HDL-c levels and higher non-HDL-c and triglyceride levels in patients with CHD.

Higher glucose levels and white blood cell counts were observed in participants with CHD, along with elevated hemoglobin levels. Regarding lifestyle factors, smoking was more common in those with CHD, while drinking status did not differ significantly between the 2 groups.

Regarding lifestyle factors, current smoking was more prevalent among participants with CHD (36.1% vs 29.0%; *P*<.001), while drinking status did not significantly differ between the groups.

In terms of comorbidities, atrial fibrillation, hypertension, diabetes mellitus, and dyslipidemia were all significantly more common in participants with CHD, as outlined in Table 1.

**Table 1.** Characteristics of study participants with and without CHD^a^ incidence (Japanese participants aged 30‐84 years, Suita Study). CHD was diagnosed by a first-ever acute myocardial infarction, sudden cardiac death within 24 hours of illness, or coronary artery disease followed by bypass or angioplasty. Values are presented as mean (SD) for continuous variables with approximately normally distribution or by median (IQR) with skewed distribution and n (%) for categorical variables. Differences in characteristics were evaluated by using the unpaired 2-tailed Student *t* test, Wilcoxon rank sums test, or chi-square test.

	CHD		*P* value
	No (n=6955)	Yes (n=305)	
Age (years), median (IQR)	55.0 (44.0-65.0)	63.0 (56.0-71.0)	<.001
Sex, n (%)			<.001
Male	3147 (45.2)	202 (66.2)	
Female	3808 (54.8)	103 (33.8)	
SBP^b^ (mm Hg), median (IQR)	123 (110-137)	138 (125-153)	<.001
DBP^c^ (mm Hg), median (IQR)	77.0 (70.0-85.0)	83.0 (74.0-89.0)	<.001
IMT_cMax^d^ (mm), median (IQR)	1.00 (0.80-1.10)	1.10 (1.00-1.30)	<.001
eGFR^e^ (mL/min/1.73 m²), mean (SD)	104 (32.2)	95.3 (63.3)	.014
BMI (kg/m²), mean (SD)	22.5 (3.10)	23.3 (3.26)	<.001
Body fat (%), mean (SD)	23.2 (6.32)	22.6 (7.06)	.15
Waist circumference (cm), median (IQR)	80.0 (73.0-86.0)	83.0 (77.0-90.0)	<.001
HDL-c^f^ (mg/dL), median (IQR)	53.0 (44.0-63.0)	46.0 (38.0-56.0)	<.001
non-HDL-c (mg/dL), mean (SD)	152 (36.9)	172 (40.5)	<.001
Triglycerides (mg/dL), median (IQR)	98.0 (70.0-143)	121 (90.0-174)	<.001
Calcium (mg/dL), mean (SD)	9.35 (0.46)	9.34 (0.43)	.61
Fructosamine (μmol/L), median (IQR)	251 (237-266)	257 (242-276)	<.001
Glucose (mg/dL), median (IQR)	95.0 (89.0-101)	100 (93.0-109)	<.001
WBC^g^ count (/mm³), median (IQR)	5.33 (4.48-6.36)	5.65 (4.81-6.78)	<.001
RBC^h^ count (10³/mm³), mean (SD)	4.53 (0.44)	4.60 (0.46)	.008
Smoking status, n (%)			<.001
Current	2019 (29)	110 (36.1)	
Past	1091 (15.7)	79 (25.9)	
Never	3845 (55.3)	116 (38)	
Drinking status, n (%)			.27
Current	3613 (51.9)	152 (49.8)	
Past	156 (2.24)	11 (3.61)	
Never	3186 (45.8)	142 (46.6)	
Atrial fibrillation, n (%)	123 (1.77)	20 (6.56)	<.001
Hypertension, n (%)	2056 (29.6)	172 (56.4)	<.001
Diabetes mellitus, n (%)	426 (6.13)	49 (16.1)	<.001
Dyslipidemia, n (%)	5280 (75.9)	265 (86.9)	<.001

a CHD: coronary heart disease.

b SBP: systolic blood pressure.

c DBP: diastolic blood pressure.

d IMT_cMax: maximum intima-media thickness of common carotid arteries.

e eGFR: estimated glomerular filtration rate.

f HDL-c: high-density lipoprotein cholesterol.

g WBC: white blood cell.

h RBC: red blood cell.

### Model Performance

The performance metrics of the 5 ML models used in our CHD prediction study provide valuable insights into their effectiveness, as shown in Table 2.

**Table 2.** Performance metrics and 95% CIs for machine learning models predicting CHD^a^ incidence (Japanese participants, aged 30‐84 years, Suita Study).

Model	Accuracy	Sensitivity	Specificity	Precision	AUC^b^	*F*_1_-score
LR^c^	0.66 (0.58‐0.75)	0.59 (0.46‐0.71)	0.74 (0.62‐0.84)	0.69 (0.55‐0.81)	0.66 (0.57‐0.75)	0.64 (0.52‐0.73)
RF^d^	0.73 (0.64‐0.80)	0.74 (0.62‐0.84)	0.72 (0.61‐0.83)	0.73 (0.61‐0.84)	0.73 (0.65‐0.80)	0.73 (0.64‐0.82)
SVM^e^	0.71 (0.62‐0.80)	0.70 (0.59‐0.81)	0.72 (0.62‐0.83)	0.72 (0.60‐0.84)	0.71 (0.63‐0.79)	0.71 (0.61‐0.80)
XGBoost^f^	0.72 (0.64‐0.80)	0.74 (0.63‐0.84)	0.70 (0.58‐0.82)	0.71 (0.60‐0.82)	0.72 (0.64‐0.80)	0.73 (0.63‐0.81)
LightGBM^g^	0.50 (0.43‐0.58)	1.00 (1.00‐1.00)	0.00 (0.00‐0.00)	0.50 (0.41‐0.59)	0.5 (0.49‐0.57)	0.67 (0.58‐0.74)

a CHD: coronary heart disease.

b AUC: area under the curve.

c LR: logistic regression.

d RF: random forest.

e SVM: support vector machine.

f XGBoost: Extreme Gradient Boosting.

g LightGBM: Light Gradient-Boosting Machine.

RF emerged as the strongest model for CHD prediction in this study, achieving the highest overall performance with an accuracy of 0.73 (95% CI 0.64‐0.80), sensitivity of 0.74 (95% CI 0.62‐0.84), specificity of 0.72 (95% CI 0.61‐0.83), and an AUC of 0.73 (95% CI 0.65‐0.80). These results highlight its balanced ability to identify both CHD and non-CHD cases effectively. In comparison, XGBoost delivered robust, yet slightly inferior, results with an accuracy of 0.72 (95% CI 0.64‐0.80), sensitivity of 0.74 (95% CI 0.63‐0.84), specificity of 0.70 (95% CI 0.58‐0.82), an AUC of 0.72 (95% CI 0.64‐0.80), and an *F*_1_-score of 0.73 (95% CI 0.63‐0.81). SVM demonstrated competitive performance, achieving an AUC of 0.71 (95% CI 0.63‐0.79), but ranked slightly behind RF and XGBoost. In contrast, LightGBM, despite its perfect sensitivity of 1.00 (95% CI 1.00‐1.00), showed a specificity of 0.00 (95% CI 0.00‐0.00) and an AUC of 0.50 (95% CI 0.49‐0.57), rendering it unsuitable for this task. LR, while serving as a baseline model, exhibited moderate performance with an accuracy of 0.66 (95% CI 0.58‐0.75), sensitivity of 0.59 (95% CI 0.46‐0.71), specificity of 0.74 (95% CI 0.62‐0.84), and an AUC of 0.66 (95% CI 0.57‐0.75), but lacked the sensitivity required for effective CHD prediction.

**Figure 1.**
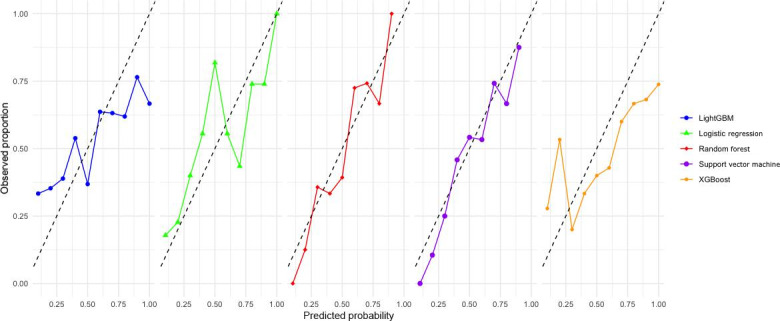
Calibration plots for machine learning models predicting CHD incidence (Japanese participants, aged 30‐84 years, Suita Study). CHD: coronary heart disease; LightGBM: Light Gradient-Boosting Machine; XGBoost: Extreme Gradient Boosting.

**Figure 2.**
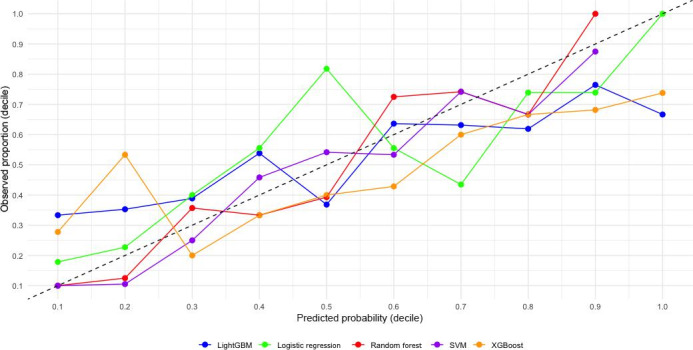
Calibration plots displaying observed-to-expected ratios for each decile of predicted CHD incidence risk (Japanese participants, aged 30‐84 years, Suita Study). CHD: coronary heart disease; LightGBM: Light Gradient-Boosting Machine; SVM: support vector machine; XGBoost: Extreme Gradient Boosting.

**Figure 3.**
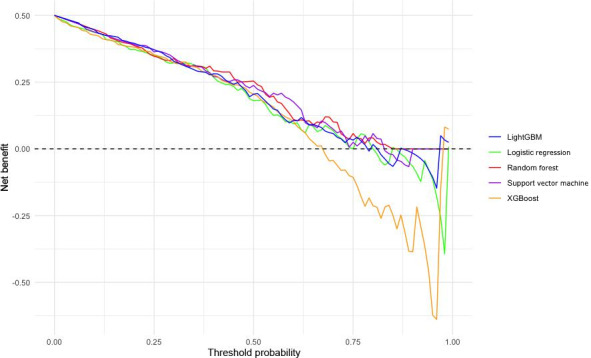
Decision curve analysis comparing machine learning models for predicting CHD incidence (Japanese participants, aged 30‐84 years, Suita Study). CHD: coronary heart disease; LightGBM: Light Gradient-Boosting Machine; XGBoost: Extreme Gradient Boosting.

The calibration curves for the 5 models (Figure 1) and the OE ratios by decile (Figure 2) provide critical insights into their predictive reliability. Among the models, RF demonstrated excellent calibration, with predicted probabilities closely aligning with observed outcomes across all deciles. This strong calibration is complemented by its performance in DCA (Figure 3).

In terms of clinical use, as illustrated in Figure 3, all models exhibit a similar positive net benefit when the threshold is below 0.5, meaning that using the predictive models is better than not using any model (treat none). However, when the threshold exceeds 0.5, the models tend to decline rapidly, with LR and XGBoost showing the most pronounced decrease, declining earlier compared to the other models.

Based on the performance metrics, RF emerges as the best model for CHD prediction in this study due to its highest overall accuracy, balanced sensitivity and specificity, strong AUC, excellent calibration, and robust clinical use across various threshold probabilities.

### Model Interpretation

**Figure 4.**
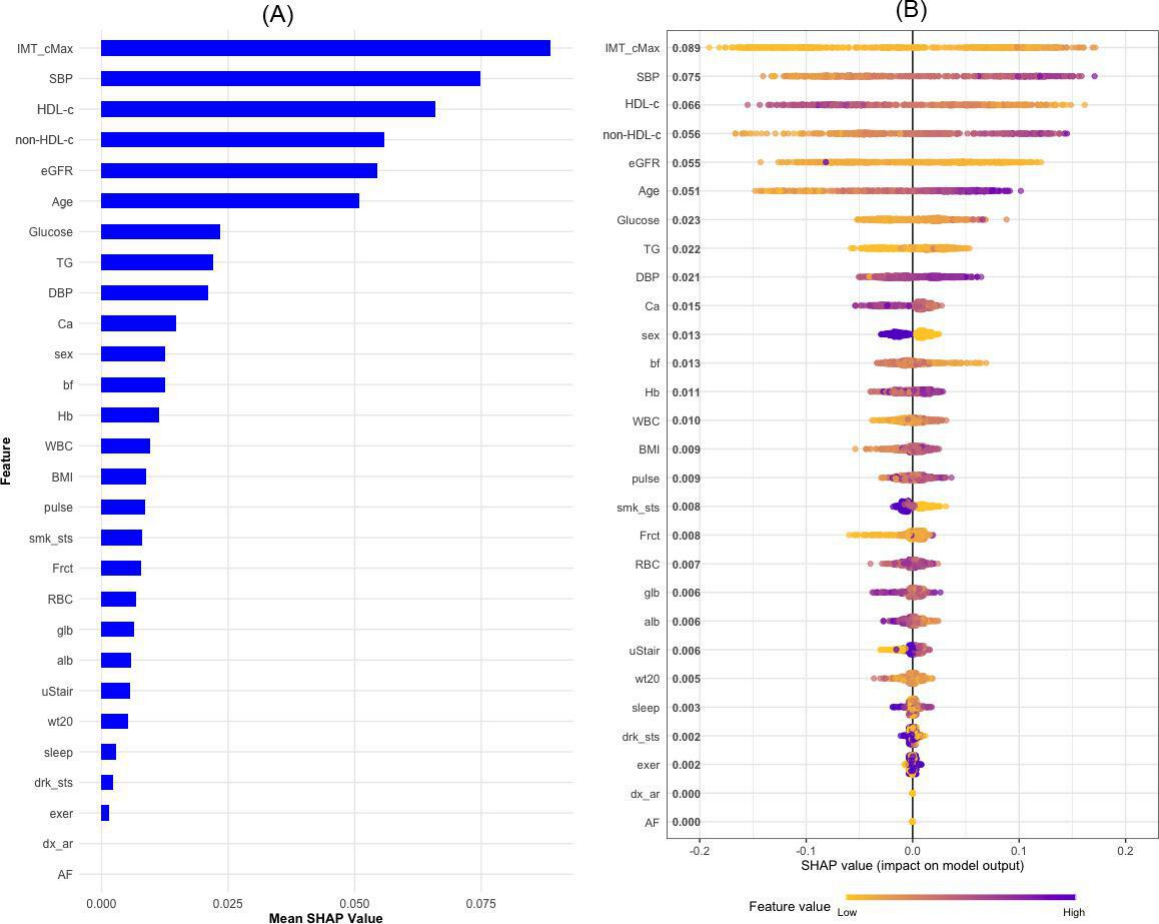
Contribution of variables to CHD incidence prediction using SHAP values (Japanese participants, aged 30‐84 years, Suita Study). (A) The bar plot shows each variable’s contribution to CHD, with bar length indicating the contribution extent. (B) The heat plot of SHAP values illustrates the relationships between variables and CHD. Purple signifies a positive relationship and yellow a negative one. Each point represents a participant, with the x-axis showing SHAP values and the y-axis indicating variable importance. bf: body fat; Ca: calcium; CHD: coronary heart disease; DBP: diastolic blood pressure; eGFR: estimated glomerular filtration rate; Frct: Fructosamine; Hb: hemoglobin; htn: hypertension; IMT_cMax: maximum intima-media thickness of common carotid arteries; HDL-c: high-density lipoprotein cholesterol; SBP: systolic blood pressure; smk_sts: smoking status; TG: triglycerides; WBC: white blood cell; wt20: weight at age of 20 years.

In Figure 4, the bar plot on the left ranks the top features contributing to CHD prediction, with IMT_cMax identified as the most influential variable, followed by systolic blood pressure (SBP), HDL-c, non-HDL-c, and eGFR. This ranking emphasizes the significance of arterial health, blood pressure regulation, lipid levels, and kidney function in assessing CHD risk. The SHAP summary heat plot on the right provides a detailed visualization of how each feature influences individual model predictions. It shows that higher values of IMT_cMax, non-HDL-c, and blood pressure are positively associated with an increased likelihood of CHD, whereas lower levels of protective factors like HDL-c and eGFR are associated with a higher risk of CHD. Other important variables, such as age, glucose levels, and triglycerides, also contribute significantly, with older age and impaired glucose regulation being linked to a higher CHD risk. Additionally, markers of inflammation like white blood cell count and other factors such as calcium levels, sex, body fat, and BMI play roles in determining CHD risk.

**Figure 5.**
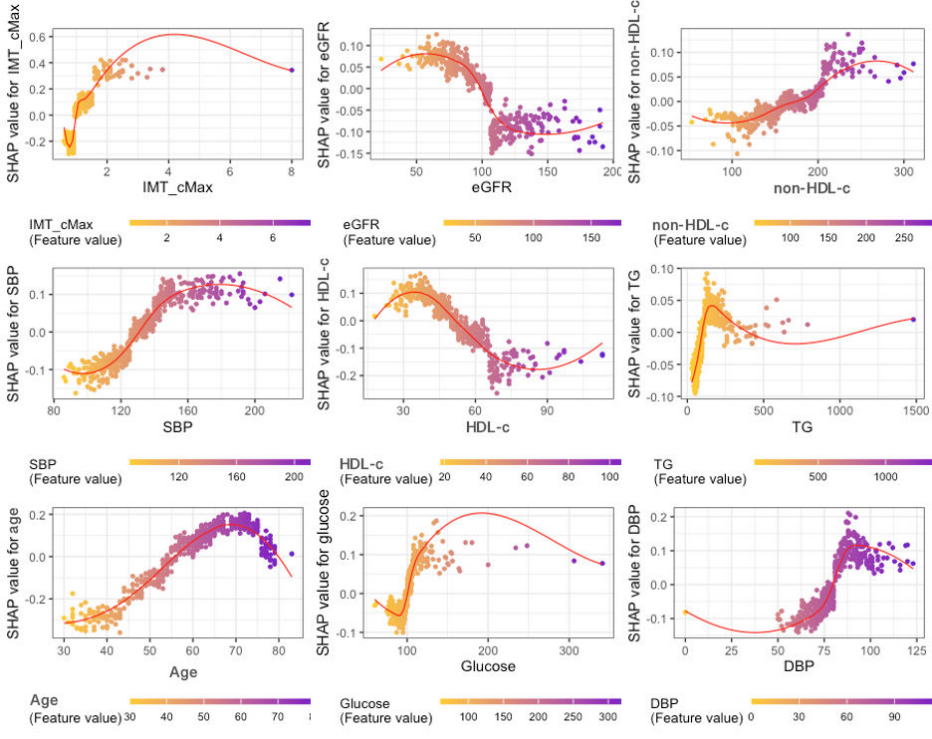
SHAP dependency plots showing the relationship between key variables and CHD risk (Japanese participants, aged 30‐84 years, Suita Study). CHD: coronary heart disease; DBP: diastolic blood pressure; eGFR: estimated glomerular filtration rate; IMT_cMax: maximum intima-media thickness of common carotid arteries; HDL-c: high-density lipoprotein cholesterol; SBP: systolic blood pressure; SHAP: Shapley Additive Explanation; TG: triglycerides.

Figure 5 consists of several SHAP dependency plots that illustrate the relationship between each key variable and CHD risk in more detail. For IMT_cMax, there is a positive association with CHD risk, showing that as the thickness of the carotid artery increases, so does the risk of CHD. The eGFR plot shows that lower eGFR values are associated with a higher risk of CHD, while higher eGFR values are associated with a lower risk, indicating the crucial role of kidney function in cardiovascular health. Non-HDL-c shows a generally positive association with CHD, where higher levels correspond to a higher risk. For SBP, the risk of CHD increases sharply with rising SBP values. HDL-c is inversely related to CHD risk, indicating its protective role, while higher triglycerides (TG) are linked to increased risk, especially at moderate levels. Age and glucose levels show a direct relationship with CHD risk, whereas older age and higher glucose levels are associated with increased risk. The SHAP value for diastolic blood pressure (DBP) also shows a positive relationship, suggesting that higher DBP levels contribute to the increased risk of CHD.

## Discussion

### Principal Findings

This study provides a comprehensive evaluation of the role of ML in predicting CHD. Among a cohort of 7260 participants, 305 were diagnosed with CHD. The analysis not only validated several well-established cardiovascular and metabolic risk factors but also identified novel predictors of CHD. Importantly, the findings underscore the use of ML models and the SHAP method in elucidating key contributors to CHD risk, with RF demonstrating superior performance, excelling in both discrimination and calibration for CHD prediction.

### Comparison With Prior Work

#### Arterial Health

Carotid IMT emerged as the strongest predictor of CHD in our study. IMT_cMax, which measures the thickness of the CCAs, is a well-established indicator of atherosclerosis and future cardiovascular events, including MI and stroke [31,32]. Multiple studies support this, showing that even a small increase in IMT correlates with a significantly elevated risk of acute MI and stroke. For instance, in the Atherosclerosis Risk in Communities study, a 0.1 mm increase in IMT corresponded to a 50% increase in CHD risk [20,31]. Therefore, measuring IMT through noninvasive techniques like ultrasound has important clinical applications in evaluating subclinical atherosclerosis and assessing CHD risk. Given that many coronary artery assessments are invasive, the use of ultrasound to measure carotid artery IMT offers a valuable alternative for early detection and risk stratification.

#### Blood Pressure, Lipid Profiles, and Glucose

SBP and hypertension were among the most critical predictors of CHD, aligning with the well-established association between elevated blood pressure and cardiovascular risk [33,34]. Both SBP and diastolic blood pressure were prominent, emphasizing the need for effective blood pressure management in reducing CHD risk [33,35].

Furthermore, non-HDL-c and triglycerides were strongly associated with CHD, confirming the importance of lipid management in cardiovascular health [36-39]. Glucose levels were also significant, suggesting that monitoring glucose metabolism is essential in cardiovascular risk management [40-42].

#### Renal Function and Metabolic Factors

The role of eGFR as a key predictor highlights the connection between renal function and CHD [43]. Impaired kidney function has been increasingly recognized as a cardiovascular risk factor, particularly due to its association with hypertension and dyslipidemia [44,45]. The results support incorporating kidney function markers in future CHD risk assessments. In addition, metabolic markers and body fat percentage were identified as important predictors, signaling the impact of obesity-related factors on cardiovascular health. These findings suggest that obesity-related measures beyond BMI should be considered in CHD risk assessments.

#### Sex

The sex-specific analysis highlighted the protective effect of being female, consistent with existing research showing that premenopausal women are generally at a lower risk of developing CHD due to protective hormonal factors [46,47]. These findings suggest the need for sex-specific strategies in managing CHD risk.

### Potential Risk Factors

One of the strengths of this study is its ability to uncover novel predictors, such as white blood cell count, which serves as a marker of systemic inflammation. Inflammation is increasingly recognized as a key player in the development of atherosclerosis and cardiovascular events. Additionally, lower calcium levels were associated with a higher risk of CHD, highlighting the importance of mineral balance in cardiovascular health. Furthermore, body fat percentage and BMI were highlighted as significant predictors of CHD, further emphasizing the need for a comprehensive evaluation of obesity-related metrics in cardiovascular risk assessments. These novel insights could lead to more personalized prevention strategies for individuals who may not exhibit classic cardiovascular risk profiles.

### Limitations

Despite the promising results, several limitations of the study need to be considered. First, the quality of the data, particularly with respect to missing values, poses a challenge. Although feature selection techniques, such as least absolute shrinkage and selection operator regression and SHAP analysis, were used to mitigate this, the impact of missing data remains a potential limitation. Second, the generalizability of the findings is limited because the study relies on a specific population. The results may not fully apply to populations with different demographic and clinical characteristics. To address this, future research should focus on evaluating these ML models in real-world clinical settings, where variability in clinical practice, missing data, and other factors may affect model performance.

### Conclusions

This study demonstrates the potential of ML in predicting CHD. The SHAP method enhances the interpretability of the prediction model, aiding health care professionals in clinical practice by supporting effective risk management and intervention strategies.

## Supplementary material

10.2196/68066 Multimedia Appendix 1 Percentage of missing data across all variables prior to imputation (Japanese participants, aged 30-84 years, Suita Study).

10.2196/68066 Multimedia Appendix 2 Correlation Coefficients for variables used in CHD Incidence prediction (Japanese participants, aged 30-84 years, Suita Study).

10.2196/68066 Multimedia Appendix 3 List of variables included in the CHD Incidence prediction model (Japanese participants, aged 30-84 years, Suita Study).

10.2196/68066 Checklist 1 TRIPOD + AI (Transparent Reporting of a Multivariable Prediction Model for Individual Prognosis or Diagnosis and Artificial Intelligence) checklist

## Abbreviations

AUC: area under the curve
CCA: common carotid artery
CHD: coronary heart disease
DCA: decision curve analysis
eGFR: estimated glomerular filtration rate
HDL-c: high-density lipoprotein cholesterol
IMT: intima-media thickness
IMT_cMax: maximum intima-media thickness of common carotid arteries
LightGBM: Light Gradient-Boosting Machine
LR: logistic regression
MI: myocardial infarction
ML: machine learning
OE: observed-to-expected
RF: random forest
SBP: systolic blood pressure
SHAP: Shapley Additive Explanation
SVM: support vector machine
TRIPOD+AI: Transparent Reporting of a Multivariable Prediction Model for Individual Prognosis or Diagnosis and Artificial Intelligence
XGBoost: Extreme Gradient Boosting
