# Supplementary figures and images for: Machine Learning Model for Predicting Coronary Heart Disease Risk: Development and Validation Using Insights From a Japanese Population–Based Study
Source: JMIR Cardio. 2025 May 12;9:e68066. doi: 10.2196/68066 (PMC12088616; doi:10.2196/68066)

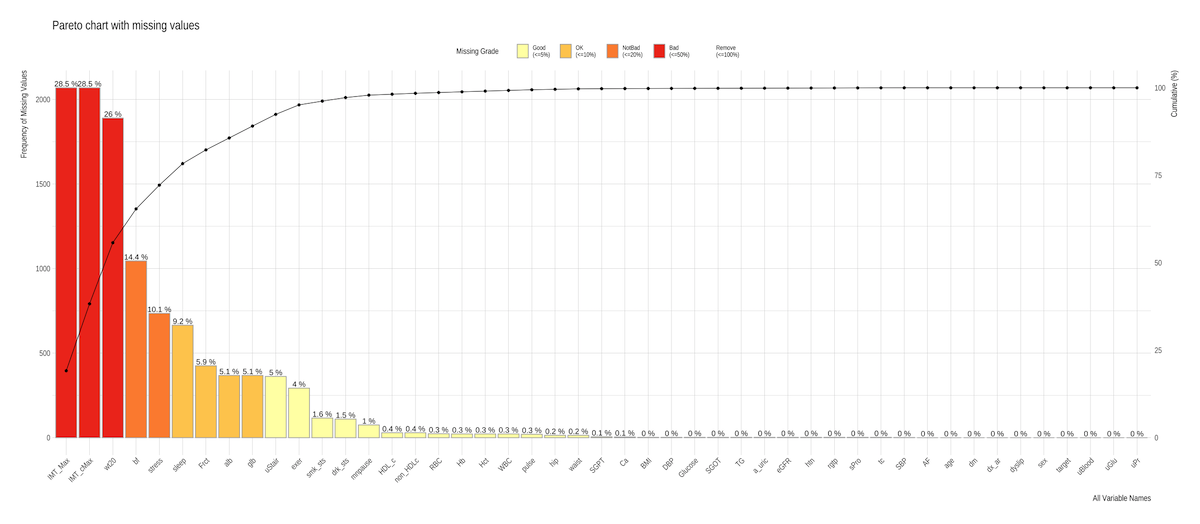

Supplement: Multimedia Appendix 1 [file cardio-v9-e68066-s001.png]

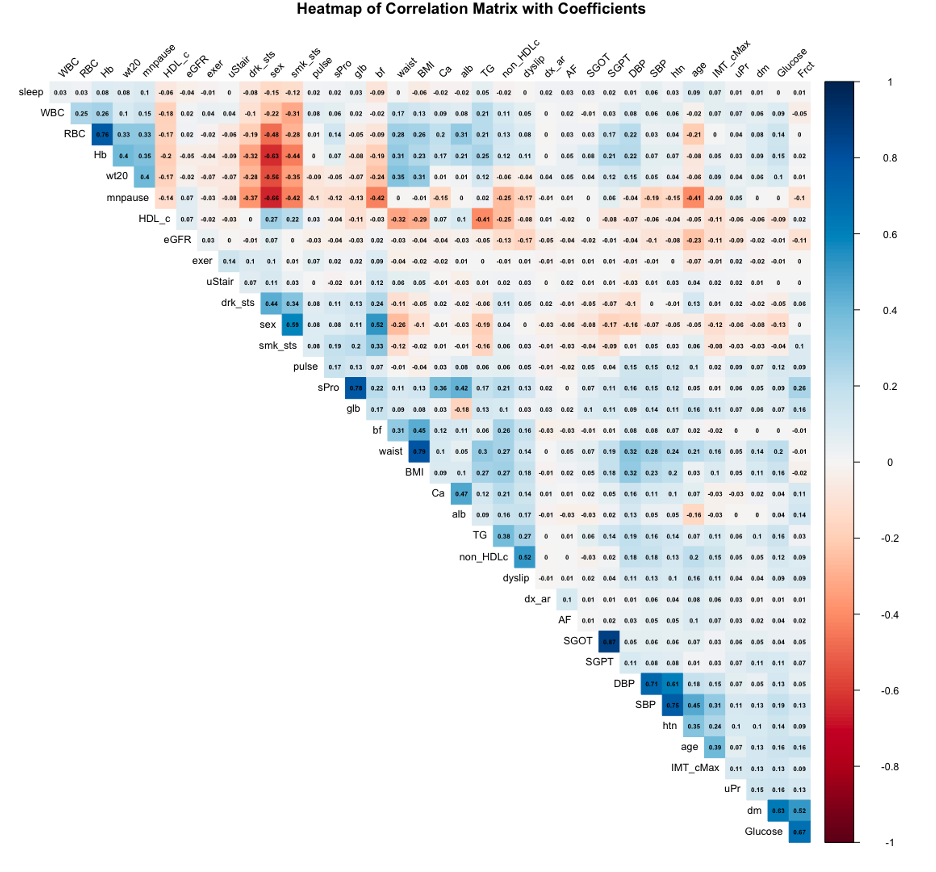

Supplement: Multimedia Appendix 2 [file cardio-v9-e68066-s002.png]
