# Supplementary material for: Machine Learning Model for Predicting Coronary Heart Disease Risk: Development and Validation Using Insights From a Japanese Population–Based Study
Source: JMIR Cardio. 2025 May 12;9:e68066. doi: 10.2196/68066 (PMC12088616; doi:10.2196/68066)
Supplement: Multimedia Appendix 3 [file cardio-v9-e68066-s003.docx]

**Table S2:** List of Variables Included in the CHD Incidence Prediction Model (Japanese Participants, Aged 30–84 Years, Suita Study).

|  | **Variable names** | **Definition** |
| --- | --- | --- |
|  | age | Age (Years) |
|  | sex | Sex (Male or Female) |
|  | pulse | Heart rate (Pulse/min) |
|  | SBP | Systolic blood pressure (mmHg) |
|  | DBP | Diastolic blood pressure (mmHg) |
|  | smk_sts | Smoking status (Current, Quit, Never) |
|  | drk_sts | Drinking status (Current, Quit, Never) |
|  | HDL-c | High density lipoprotein cholesterol (mg/dL) |
|  | TG | Triglycerides (mg/dL) |
|  | non-HDL-c | Non-high density lipoprotein cholesterol (mg/dL) |
|  | Glucose | Blood glucose (mg/dL) |
|  | Alb | Albumin (g/dL) |
|  | Ca | Serum calcium (mg/dL) |
|  | Frct | Fructosamine (μmol/L) |
|  | BMI | Body mass index (kg/m2) |
|  | wt20 | Weight at age 20 (kg) |
|  | bf | Body fat percentage (%) |
|  | uStair | Use of stairs (Stair use 1. ≥ 80%, 2. 6-80%, 3. 4-60%, 4. 2-40%, 5. < 20%) |
|  | exer | Exercise (1: Yes, 0: No) |
|  | RBC | Red blood cell (×103 /mm3) |
|  | Hb | Hemoglobin (g/dL) |
|  | WBC | White blood cell (/mm3) |
|  | IMT_cMax | the maximum intima-media thickness of common carotid arteries (mm) |
|  | Uric acid | Uric acid (mg/dL) |
|  | sleep | Time of sleeping (1. ≤ 6hr, 2. 7hr range, 3. 8hr range, 4. ≥ 9hr, 5. Unknown, shift system) |
|  | AF | Atrial Fibrillation (1: Yes, 0: No) |
|  | dx_ar | Arrythmia (except AF) (1: Yes, 0: No) |
|  | eGFR | Estimated Glomerular filtration rate (mL/min/1.73m2) |
